# Effect of low-dose intravenous dexamethasone on post lumbar discectomy pain: randomized and double blind study

**Published:** 2012-11

**Authors:** Amirhossein Haghir, Firouz Salehpour, Ali Meshkini, Reza Vafaei, Asghar Ashrafi Hafez

**Affiliations:** ^*a*^Imam Reza Teaching Hospital, Tabriz University of Medical Sciences, Tabriz, Iran.; ^*b*^Shahid Beheshti University of Medical Sciences, Tehran, Iran.

## Abstract

**Background:**

The Pain is the most common complaint in various diseases. Postoperative pain is common complication and spatially in elderly patient because of exacerbation of heard and vessel was impotents. The aim of this study was evaluation of the effect of low-dose IV dexamethasone on postoperative pain in patients with lumbar discectomy.

**Methods:**

In a clinical trial that studied in neurosurgery wards of Shohada and Imam Reza hospitals affiliated to the Tabriz University of Medical Sciences on patients underwent lumbar discectomy, the effect of low-dose IV dexamethasone on postoperative pain was evaluated. 80 patients divided in 2 equal groups, we used IV morphine (present routine treatment) in group A and IV morphine in addition to 8mg IV dexamethasone in group B, for reducing post lumbar discectomy pain.

**Results:**

21 patients in group A, & 22 in group B were male and 19 patients in group A & and 18 in group B were female (P=0.823). Mean age of patients in groups A and B was 39.32 and 39.22 years, respectively (P equal to 0.945). Mean of pain score (VAS) at 6 hours post-operation in group A and B was 6.97 and 6.75, respectively (P equal to 0.065). VAS at 12, 18 or 24 hours post-operation in both groups didn’t differ significantly (P greater than 0.05).

**Conclusions:**

We didn't observe any significant reduction in post lumbar discectomy pain after adding 8 mg dexamethasone into morphine. Regarding other studies, it seems that higher doses of dexamethasone should be used to achieve a significant pain reduction.

**Keywords:**

Lumbar discectomy, Dexamethasone, Postoperative pain, Pain measurement


					Volume 4, Suppl. 1 Nov 2012
Publisher: Kermanshah University of Medical Sciences
URL: http://www.jivresearch.org
Copyright:
Congress of Iranian Neurosurgeons, 14-16 November 2012, Kermanshah, Iran
Paper No. 51
Effect of low-dose intravenous dexamethasone on post lumbar
discectomy pain: randomized and double blind study
Amirhossein Haghir a
, Firouz Salehpour a
, Ali Meshkini a,*
, Reza Vafaei b
, Asghar Ashrafi Hafez b
a
Imam Reza Teaching Hospital, Tabriz University of Medical Sciences, Tabriz, Iran.
b
Shahid Beheshti University of Medical Sciences, Tehran, Iran.
Abstract:
Background: The Pain is the most common complaint in various diseases. Postoperative pain is common complication
and spatially in elderly patient because of exacerbation of heard and vessel was impotents. The aim of this study
was evaluation of the effect of low-dose IV dexamethasone on postoperative pain in patients with lumbar
discectomy.
Methods: In a clinical trial that studied in neurosurgery wards of Shohada and Imam Reza hospitals affiliated to the
Tabriz University of Medical Sciences on patients underwent lumbar discectomy, the effect of low-dose IV
dexamethasone on postoperative pain was evaluated. 80 patients divided in 2 equal groups, we used IV morphine
(present routine treatment) in group A and IV morphine in addition to 8mg IV dexamethasone in group B, for
reducing post lumbar discectomy pain.
Results: 21 patients in group A, & 22 in group B were male and 19 patients in group A & and 18 in group B were
female (P=0.823). Mean age of patients in groups A and B was 39.32 and 39.22 years, respectively (P equal to
0.945). Mean of pain score (VAS) at 6 hours post-operation in group A and B was 6.97 and 6.75, respectively (P
equal to 0.065). VAS at 12, 18 or 24 hours post-operation in both groups didn't differ significantly (P greater than
0.05).
Conclusions: We didn't observe any significant reduction in post lumbar discectomy pain after adding 8 mg
dexamethasone into morphine. Regarding other studies, it seems that higher doses of dexamethasone should be used
to achieve a significant pain reduction.
Keywords:
Lumbar discectomy, Dexamethasone, Postoperative pain, Pain measurement
* Corresponding Author at:
Ali Meshkini : Associate Professor of Neurosurgery, Imam Reza Teaching Hospital, Tabriz University of Medical Sciences, Tabriz, Iran,
Email: meshkinia@yahoo.com, (Meshkini A.).

				

